# Large-scale sequestration of atmospheric carbon via plant roots in natural and agricultural ecosystems: why and how

**DOI:** 10.1098/rstb.2011.0244

**Published:** 2012-06-05

**Authors:** Douglas B. Kell

**Affiliations:** 1 School of Chemistry and Manchester Interdisciplinary Biocentre, University of Manchester, 131 Princess St, Manchester M1 7DN, UK; 2 Biotechnology and Biological Sciences Research Council, Polaris House, North Star Avenue, Swindon, Wiltshire SN2 1UH, UK

**Keywords:** soil, carbon, sequestration, systems biology, breeding

## Abstract

The soil holds twice as much carbon as does the atmosphere, and most soil carbon is derived from recent photosynthesis that takes carbon into root structures and further into below-ground storage via exudates therefrom. Nonetheless, many natural and most agricultural crops have roots that extend only to about 1 m below ground. What determines the lifetime of below-ground C in various forms is not well understood, and understanding these processes is therefore key to optimising them for enhanced C sequestration. Most soils (and especially subsoils) are very far from being saturated with organic carbon, and calculations show that the amounts of C that might further be sequestered (http://dbkgroup.org/carbonsequestration/rootsystem.html) are actually very great. Breeding crops with desirable below-ground C sequestration traits, and exploiting attendant agronomic practices optimised for individual species in their relevant environments, are therefore important goals. These bring additional benefits related to improvements in soil structure and in the usage of other nutrients and water.

## 1. Introduction: why sequester atmospheric CO_2_?

It is well known that atmospheric CO_2_ is increasing at some 2 ppmv p.a., mainly as a result of human activities such as fossil fuel combustion, and that this has taken its values from *ca* 280 ppmv at ‘pre-industrial’ levels to more like 390 ppmv today [1]. To avoid the predicted increases in global temperature contingent upon ‘greenhouse gas’ effects we need not only to lower the emissions but preferably to find means of sequestering atmospheric CO_2_ over extended periods. Similar arguments apply to all other greenhouse gases [2], such as CH_4_ and N_2_O.

**Figure 1.**
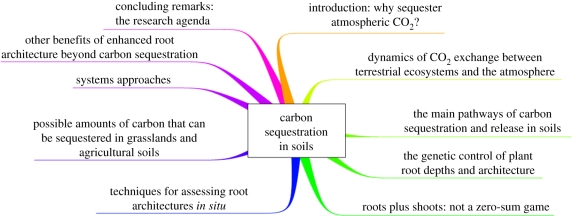
A ‘mind map’ [5] setting out the contents of the paper. To read it start at ‘12 o'clock’ and read clockwise.

On geological time-scales, atmospheric CO_2_ levels were much (possibly 10-fold) greater than they are now [3], and the main means by which sequestration of atmospheric carbon was achieved, especially in the Devonian, Carboniferous and Cretaceous eras, was through plant photosynthesis. The question obviously arises as to whether we can drive such improved sequestration in the modern era in useful quantities and at useful rates. I believe that we can [4]. An overview of the article is given as a ‘mind map’ [5] in figure 1.

## 2. Dynamics of CO_2_ exchange between terrestrial ecosystems and the atmosphere

The first point to make is that terrestrial ecosystems including soils globally hold at least twice as much carbon (*ca* 1500–2500 Pg/Gt) as does the atmosphere (750 Pg) [6,7], so an overall increase in soil carbon by 10% implies (crudely) a decrease in (or more accurately a saving of an increase in) atmospheric carbon of at least 20%. (Note that at 750 Pg ≈375 ppmv atmospheric CO_2_, 2 Pg of C removed from or not added to the atmosphere ≈1 ppmv removed or not added.) Overall fluxes to and from the soil are substantial, probably 60 Pg yr^−1^ or more, albeit low in comparison to these pools [7–9]. This means that determining even the net direction of CO_2_ transfer requires comparatively high-precision measurements [10]. Nevertheless, reasonably accurate estimates of net fluxes of ±1–2 t (ha yr)^−1^ are easily attainable. Present agricultural ecosystems are rather depleted of soil carbon [11–14], and the existing ‘sink’ in such soils could certainly accommodate 50 t ha^−1^. Given that world cropland and grassland each account for some 2300 Mha [15], the scope for increased sequestration in terrestrial ecosystems is clearly substantial [4], and—as with the exploitation of solar energy [16]—something we are very far from saturating.

It also needs to be recognised that there has been a certain dichotomy between most studies, that have been designed to analyse natural ecosystems—‘what is there’—and what could be done if we chose to breed and deploy suitable plants whether as food or non-food crops—‘what might be’ [4]. I shall tend to use the former as an existence proof of possibilities, while recognising that it is the latter that is the real goal.

Finally, here I note that the atmosphere is also in contact with the oceans [17] (and in pseudo- but not full equilibrium with them; if it were, the annual oscillations would tend to be much more heavily damped), and that the oceans sequester some 38 000 Pg C or 50 times that in the atmosphere [7,18]. This means that any eventual tendency to decrease atmospheric CO_2_ effected by C sequestration in soil can be balanced by degassing of CO_2_ from the oceans, so that what we are talking about here is stabilising values at their present levels rather than reducing them substantially (I thank Gideon Henderson for a useful discussion on this point). The liming of oceans may also offer some important opportunities there [19] (and see http://www.cquestrate.com/).

## 3. The main pathways of carbon sequestration and release in soils

There are four main steps in a systems biology approach to understanding complex networks [20,21]. Steps 1 and 2 are essentially qualitative, and define the steps and the interacting partners (sources and sinks for each step), and whether such interactions are direct and stoichiometric or indirect and ‘regulatory’. Each ‘step’ may of course consist of multiple substeps. The third and fourth stages provide any known (or ‘generalised’ [22]) kinetic rate equations and the values of their parameters. Interoperable standards exist for describing such networks in XML [23], as well as for their graphical representation [24]. Armed with such information, it is then possible to develop a stochastic or ordinary differential equation model of the entire system of interest, whether based on ‘lumped’ compartments or involving explicit spatial differentiation.

**Figure 2.**
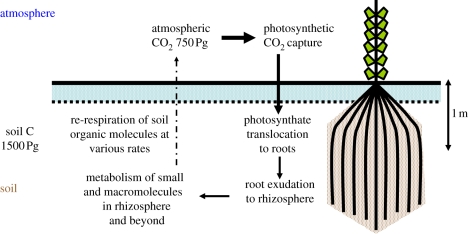
High-level analysis of the major processes involved in soil carbon sequestration for photosynthetically fixed CO_2_.

In the spirit of step 1, figure 2 illustrates in general terms (for a more detailed version, see [25]) the main processes in soil operating to capture, sequester, transform and (in time) re-release atmospheric CO_2_. The main initial step is necessarily photosynthetic CO_2_ capture, followed by its translocation below ground into plant roots [26]. Partly under genetic, nutritional and hormonal control, roots can extend to varying depths, and thereby deposit carbon as root biomass. Probably more important is the fact that roots exude all kinds of carbon-containing components into the rhizosphere [27], a complex and imperfectly characterised zone containing numerous microbes (including mycorrhiza [28,29]). From here, further transformations [30] can produce a variety of carbon-containing small and macromolecules that can exist in soil [31] and contribute to the soil structure (not least by aggregating inorganic soil particles [11,32]). Depending on the nature of the molecule and other conditions such as pH, water activity and dissolved oxygen tension, such ‘carbon’ will reside in soil for a greater or lesser period (defining its ‘recalcitrance’ [33]). Interestingly, it is increasingly being recognised (e.g. [34,35]) that this recalcitrance may be more a property of where the molecule is sequestered than what it is chemically, and may also depend on supplies of fresh carbon [36]. At all events, eventually, most of the carbon will be re-respired to the atmosphere as CO_2_. As a systems property [37], clearly the steady-state extent of sequestration depends on the topology and kinetics of all steps in the network, with the control of flux being distributed (e.g. [20,38]). Equally clearly, the relative contributions of different steps will vary in different soils [33]. However, as the step that determines the initial distribution of carbon in the soil by plant roots [26,39], it is the rooting process itself—the focus of this themed issue—on which we necessarily concentrate.

## 4. The genetic control of plant root depths and architecture, and G X E interactions

As mentioned, plant root depths and architecture are partly controlled by physical and agronomic (and hormonal, e.g. [40,41]) factors, but to a substantial degree [4,42–46] it is the genetic make-up of the organism (including genes whose products affect hormone production and distribution [47]) that determines how deep and bushy its roots can become. Some plants can indeed produce very substantial root architectures (e.g. [48–51], and there is evidence for genetically determined variation in root architecture between plant types (e.g. [48,49,52]), between different cultivars of the same plant (e.g. [53–62]), and between different mutant strains with known genetic alleles or defects (e.g. [63–73]). In some cases, the number of genes involved in effecting substantial morphological changes may be quite small (e.g. [74,75]).

Clearly this encourages us to develop breeding programmes for plants with improved root architectures that can sequester carbon (and other nutrients, plus water) more effectively [4]. Such encouragement should be seen in broad terms as a contribution to ecosystem services [76], as well as agricultural yields [55,77], and the economic benefits derived therefrom might be enhanced by the payment of carbon credits [14,15,78–80].

I stress the breeding aspects, since we now know, especially from work with animals, that ‘genomic selection’ can speed genetic gain considerably [81–89], and it will soon be the norm to exploit modern whole-genome sequencing methods [90,91] to sequence every organism of interest in a breeding population.

This said, the necessary breeding will need to be assessed under a variety of agronomic conditions, since there is little doubt that agronomic practices can have a considerable impact on plant yields (there is substantial variation in yields across individual fields planted with the same crop, e.g. [92]), and in a manner that is of course dependent on the genetic make-up of the plants (G×E interactions). The System of Rice Intensification (e.g. [93–98]) provides a particularly nice example of that.

## 5. Roots plus shoots: not a zero-sum game

It is sometimes opined that any breeding-based improvement (i.e. increase) in below-ground biomass would be balanced by an equivalent decrease in above-ground (and hence agriculturally harvestable) biomass. This is *a priori* implausible since they are more likely to feed each other than not, and most bioprocess fluxes are in fact demand-led [99]. At all events, there is plenty of evidence that the distribution of resource between root and shoot is not a zero-sum game:

— larger plant types as judged by above-ground biomass do in general tend to have larger roots—compare trees and typical crop plants, for instance [100,101];
— many mutants that have larger roots have above-ground biomass that is not smaller, and is often larger, than those of their parental wild type or ‘baseline’ strain (e.g. [45,77,102–112]);
— simple improvements in agronomic practices such as appropriate nutrient supply can increase the total amount of both root and shoot biomass (e.g. [113–117]), and in systems such as the System of Rice Intensification mentioned above apparently quite substantially so;
— similar behaviour (simultaneous increases in root and shoot biomass) can be induced by other non-host-genetic means that do not directly involve nutrients [118–120].

It is therefore entirely reasonable that we can improve plant root traits (and specifically to increase the size and extent of roots) in a manner that is not at the cost—and in many cases will likely be to the benefit—of above-ground traits including harvestable biomass. Both genetic and environmental (agronomic) approaches are likely to be of benefit here.

## 6. Techniques for assessing root architectures *in situ*

Science consists of both analysis and synthesis, and while high-throughput genomics has of course increased its throughput massively over the last decade, the same cannot be said of phenotyping [121]. Traditional (and many modern) methods for assessing the extent and nature of root phenotypes involve careful excavation and recording (e.g. [56,58,62,122]), but we need automated, non-invasive methods that likely involve some kind of spectroscopy or imaging [123] coupled to sophisticated computation. All have strengths and weaknesses, and some may be surrogates that measure properties (e.g. moisture content, or the force required to remove a plant from the soil [96]) that simply correlate with root properties, but some instrumental methods that appear promising include methods based on various kinds of impedimetry / capacitance / permittivity [124–128] (see also [129,130]) and impedance tomography [131], optical imaging [59,132–134], X-ray microtomography [57], ground-penetrating radar [131,135,136], microwave spectroscopy [137], neutron spectroscopy and tomography [138] and magnetic resonance imaging [139] (that may be combined with positron emission tomography [140]). Fusion methods that combine multiple inputs can always [141] be expected to perform better than individual approaches.

## 7. Possible amounts of C that can be sequestered in grasslands and agricultural soils

Calculations suggest (http://dbkgroup.org/carbonsequestration/rootsystem.html) [4] that the amount of C that can be stored in agricultural soils is considerably greater than is stored there now [12,14,142,143], namely in amounts similar to those that might be generated anthropogenically for the next 50 years, thereby stabilising atmospheric CO_2_ at present levels. However, it is to be recognised that these calculations carry considerable uncertainty [144] as we know comparatively little about the rate and extent of root growth and in particular (e.g. [145–148]) the lifetime(s) of the various soil components before ultimately they are re-respired. The variation in sequestration time (‘recalcitrance’) of different forms of carbon-containing molecule can be very great, implying scope for increasing it by selective breeding (much as one can breed for enhanced degradability when this is desired for biomass crops [149]). Some analyses of existing grasslands and energy crops imply that at least 100 t ha^−1^ of C may be sequestered in roots (or at least below ground) in the steady state [150,151], while tree forests usually sequester even more [152,153] (so deforestation [153,154] and forest drought [155] are especially damaging). One metre depth of soil containing just 1 per cent C at a bulk relative density of unity equates to 10 kg m^−2^ or 100 t ha^−1^, so estimates of 200 t ha^−1^ in just the top metre alone [151] imply a considerably greater carrying capacity than that presently sustained, even before more recalcitrant forms of carbon such as biochar [156–158] are considered.

## 8. Other benefits of enhanced root architectures beyond C sequestration

This article has concentrated on the benefits to be had from improved root architectures largely in terms of carbon sequestration. However, it would be remiss not to stress that such improved root architectures also bring many other agricultural benefits [55,77], including improvements to soil structure [159], hydrology [160], drought tolerance (e.g. [161,162]) and N use efficiency [163]; in some cases these benefits may well prove to be more important overall, but can certainly be seen as additional benefits with regard to the C sequestration agenda.

## 9. Systems approaches

As mentioned above, it is necessary to consider the whole (eco)system when undertaking studies of this type, and a strategy or intervention that seems to have a proximate benefit may have an ultimate disbenefit (or vice versa), due to ignoring important contributors to net balances or the propagation of the change via complex positive and negative feedback loops (e.g. [25], and for a more general account [164]). Thus changes in climate and raised CO_2_ may have unexpected effects on root–soil interactions [165] or any other processes, and the ability to sequester C will depend not only on the amount, extent and recalcitrance of plant roots but of the production rate and nature of root exudates, the amount of nitrogen [166] and other nutrients, and biophysical properties such as moisture content, soil compaction and the like. We also need to be ever mindful that changes affecting below-ground processes, especially if conditions are allowed to become anaerobic, might turn fixed CO_2_ into much more damaging gases such as methane and nitrous oxide [2]—something to be avoided at all costs.

## 10. Concluding remarks: the research agenda

This brief review purposely takes a relatively restricted and high-level approach to the problem of sequestering atmospheric carbon in soils. It recognises that (i) soils contain much carbon but are far from saturated with regard to organic matter, (ii) most soil carbon is derived from roots rather than from shoots and leaf litter, (iii) much of the carbon and most of the measurements thereof are restricted to the top 1 m of soil, and developing plants with 2 m roots could sequester considerably more C than is done presently, (iv) the transformation pathways and lifetimes of carbon components in the soil (both topsoil and subsoils), and what determines them both biologically and biophysically, are much less well understood than we would like, (v) the longer any particular form of carbon is held below ground before it is re-respired or emitted, the greater the amount that can be sequestered in the steady state, (vi) many analyses have concentrated more on ‘what is there’ than ‘what we might do about it’, and (vii) modern whole genome sequence-driven breeding offers huge opportunities for accelerating plant improvement.

As with scientific advances generally [167], we may expect to see iterative cycles, in that we may find empirically (through studying the variance between experiments [168]) that a particular cultivar treated with a particular agronomy does well with regard to soil carbon sequestration, and we may find from phenotypic (including ‘omics’) measurements that roots are involved mechanistically. We might then seek to apply directed breeding and agronomic practices that improve such root properties directly and then test if such crops also sequester carbon more effectively.

Thus, molecular breeding and appropriate agronomy (largely still matters for experiment), coupled to the necessary phenotyping approaches, especially non-invasive measurements of various kinds plus the attendant informatics and improved modelling [169], can lead to improved food and non-food crops that also have desirable carbon sequestration traits. Consequently, there is much to play for.
